# Organic–Inorganic Hybrid Materials II: Some Additional Contributions to the Topic

**DOI:** 10.3390/polym13152390

**Published:** 2021-07-21

**Authors:** Jesús-María García-Martínez, Emilia P. Collar

**Affiliations:** Polymer Engineering Group (GIP), Polymer Science and Technology Institute (ICTP), Spanish Council for Scientific Research (CSIC), C/Juan de la Cierva, 3, 28006 Madrid, Spain

By following the successful editorial pathway of the recently published former Special Issue dedicated to Organic–Inorganic Hybrid Materials [1,2], this second part offers a number of new original contributions and/or approaches to the topic. Once again, these new investigations matched the definition of hybrid material as per the IUPAC (International Union of Pure and Applied Chemistry) recommendations [3]. That means a hybrid material is composed of an intimate mixture of inorganic components, organic components, or both types of component, which usually interpenetrate on scales of less than 1 μm [3].

The latter implies that this definition can be identified with a myriad of industrial and/or academic approaches. In fact, when applied to organic–inorganic hybrid materials open plenty of research and design lines for material scientists. It is noteworthy that the organic–inorganic hybrid materials are multi-component compounds with at least one of the parts in the sub-micrometric and/or the nano-metric domain [4].

Additionally, the literature usually classifies the organic–inorganic hybrid systems into two families or classes (Class-I and Class-II), attending to the type of interactions between the phases (weak or strong, respectively) [5]. Therefore, Class-I organic–inorganic hybrid compounds imply weak interactions such as Van der Waals, hydrogen bonds, electrostatic, etc.; conversely, Class-II compounds have a real chemical bond between phases. The third option is a combination of the two types of interactions in the same hybrid system [6]. In essence, it can be said that the organic–inorganic materials are multi-component compounds with at least one of their organic (the polymer) or inorganic components in the nano-metric-size domain, which confers the material as a whole of greatly enhanced properties respecting the constitutive parts in isolation [4,5,6,7].

Under these premises, this additional Special Issue complements the previous one [1,2] with five new approaches, covering a wide range of conceptual and application spectra.

Therefore, the first article by Figueira et al. [8], dedicated to the memory of one of the co-authors (Professor Carlos. J. R. Silva), is devoted to the study of the synthesis and characterization of five new organic–inorganic hybrids (OIH) sol-gel materials obtained from a functionalized siloxane 3-glycidoxypropyltrimethoxysilane (GPTMS) by the reaction with a new Jeffamine®, a secondary diamine, (SD-2001), and a triamine, (T-403). The resulting materials (OIH sol-gel materials) were studied and characterized by UV-visible absorption spectro-photometry, steady-state photoluminescence spectroscopy, and electrochemical impedance spectroscopy. From there, it was found that the optical and electrical properties of the obtained new OIH materials exhibited promising properties as support films in an optical sensor area. Additionally, some investigations were developed on the chemical stability of the OIH materials in contact with cement pastes after three different periods of time, concluding that many of the new materials exhibited improved behaviors which were very promising in the alkaline environments caused by the cement paste. This fact makes these materials to very promising candidates for their potential application as a support film in optical fiber sensors in the area of civil engineering.

The work by Alsame et al. [9] is focused on an ease hydrothermal synthesis of polyaniline (PANI)-modified molybdenum disulfide (MoS2) nanosheets employed to obtain a novel 3D organic–inorganic hybrid material. An exhaustive characterization of the obtained material by using field emission scanning electron microscopy, high-resolution transmission electron microscopy, energy-dispersive X-ray spectroscopy and X-ray diffraction studies led the authors to conclude that the obtaining procedure of the organic–inorganic hybrid material (PANI–MoS_2_) was successful. Additionally, this organic–inorganic hybrid material has demonstrated its efficiency in the extraction and pre-concentration of trace mercury ions, thanks to the selective complexation between the sulfur ion of PANI–MoS_2_ with Hg(II)—the lowest concentration threshold being as little as 0.03 µg L^−1^—being applied to the proper analysis of real samples. This suggests that the PANI–MoS_2_ hybrid material can be used for trace Hg(II) analyses for environmental water monitoring.

The design of a flat-sheet direct-contact membrane distillation (DCMD) module to remove nitrates from water is the purpose of the investigation by Orroji, Razmjou, and Ebrahimi [10]. For such a purpose, a polyvinylidene fluoride (PVDF) membrane was employed in a DCMD process performed at 1 atm and below 100 °C. It was found that, during the entire process, the electrical conductivity of the feed containing nitrate increased, while the permeation remained constant, resulting in the fact that the nitrate ions did not pass through the membrane while pure water is obtained. In order to have a better hierarchical surface control, the authors proposed modifying the membrane with TiO_2_ nano-particles for multi-layer roughness on the micro/nano-scale domains. Further, the incorporation of 1H,1H,2H,2H-Perfluorododecyltrichlorosilane (FTCS) to the TiO_2_-modified surface of the membrane with the purpose to obtain super-hydrophobic properties improving its separation performance, was also explored. Finally, the authors compared types of membranes by the experimental, pilot-scale, and simulation procedures, and found very interesting results and predictions about the surface needed for a proper separation process.

The work contributed by Ahmad et al. [11] focused on the kinetics of photo-isomerization and the time evolution of hybrid thin films, but with a novel insight approach based on considering the azo-dye methyl red (MR) group linked into graphene embedded onto a polyethylene oxide matrix (PEO), forming an organic–inorganic hybrid material, PEO-(MR-Graphene). The kinetics study was undertaken by UV-Vis and FTIR spectroscopy. Additionally, they used new models developed with new analytical methods. The authors reported that the presence of azo-dye MR in the compound is the key aspect for the resource action of the trans to cis cycles (and vice versa) through UV to visible-illumination relaxations. Consequently, the authors found that these hybrid composite thin films may be used as applicants for photochromic molecular switches, light-gated transistors, and molecular solar thermal energy storage media.

Finally, and because the properties of hybrid silica xerogels obtained by the sol-gel method are strongly dependent on both the precursor and the synthesis conditions, Espinal-Viguri and Garrido et al. [12] investigate the influence of the organic substituent of the precursor on the sol-gel process conditioning the final structure of the obtained materials. The latter applies to the xerogels with tetraethyl orthosilicate (TEOS) and alkyltriethoxysilane or chloroalkyltriethoxysilane at different molar percentages (RTEOS and ClRTEOS, R = methyl [M], ethyl [E], or propyl [P]). The authors conclude that the intermolecular forces caused by the organic groups jointly to the chlorine atom in the precursors were ascertained by comparison of the sol-gel process between alkyl and chloroalkyl series. Therefore, the authors conclude that the alkyl chain and the chlorine atom of the precursor in these materials determine their inductive and steric effects on the sol-gel process. Additionally, the authors studied the microstructure of the obtained xerogels by deconvolution methods to Fourier-transformed infrared spectra. Therefore, the distribution of (SiO)_4_ and (SiO)_6_ rings into the silicon matrix of the xerogels is revealed. These results are fully consistent with X-ray diffraction spectra, indicating that the local periodicity associated with four-fold rings increases the amount of the precursor. To conclude, the authors found that the combination of the sol-gel process and the emerging ordered domains determine the final structure of the hybrid materials and, therefore, their properties and potential applications.

To summarize, in the light of the present and former [1,2] Special Issues, it can be affirmed that the topic of organic–inorganic hybrid materials covers a broad spectrum of approaches and/or applications. In any case, it is the inter-phase between the components which plays a crucial role in these final performances of the hybrid materials. This fact becomes the critical aspect when intend to obtain this type of hybrid materials with “tailor-made” organized structures in every one of the nano-, meso-, micro- and meso-scale factors ultimately involved.

We would like to thank all the authors for their contributions and encourage them to continue enthusiastically with their research.
